# Supplementary material for: Arabidopsis TCP4 transcription factor inhibits high temperature-induced homeotic conversion of ovules
Source: Nat Commun. 2023 Sep 13;14:5673. doi: 10.1038/s41467-023-41416-1 (PMC10499876; doi:10.1038/s41467-023-41416-1)
Supplement: Supplementary file 1 — Supplementary Information [file 41467_2023_41416_MOESM1_ESM.pdf]

Supplementary Information

Arabidopsis TCP4 transcription factor inhibits high temperature-induced homeotic conversion of ovules

Jingqiu Lan[1,2,3], Ning Wang[1,3], Yutao Wang[1], Yidan Jiang[1], Hao Yu[1], Xiaofeng Cao[2], Genji Qin[1*]

[1]State Key Laboratory of Protein and Plant Gene Research, School of Life Sciences, Peking University, Beijing 100871, China.

[2]State Key Laboratory of Plant Genomics and National Center for Plant Gene Research, CAS Center for Excellence in Molecular Plant Sciences, Institute of Genetics and Developmental Biology, Chinese Academy of Sciences, Beijing 100101, China.

[3]These authors contributed equally to this work: Jingqiu Lan, Ning Wang.

[*]Corresponding author: qingenji@pku.edu.cn

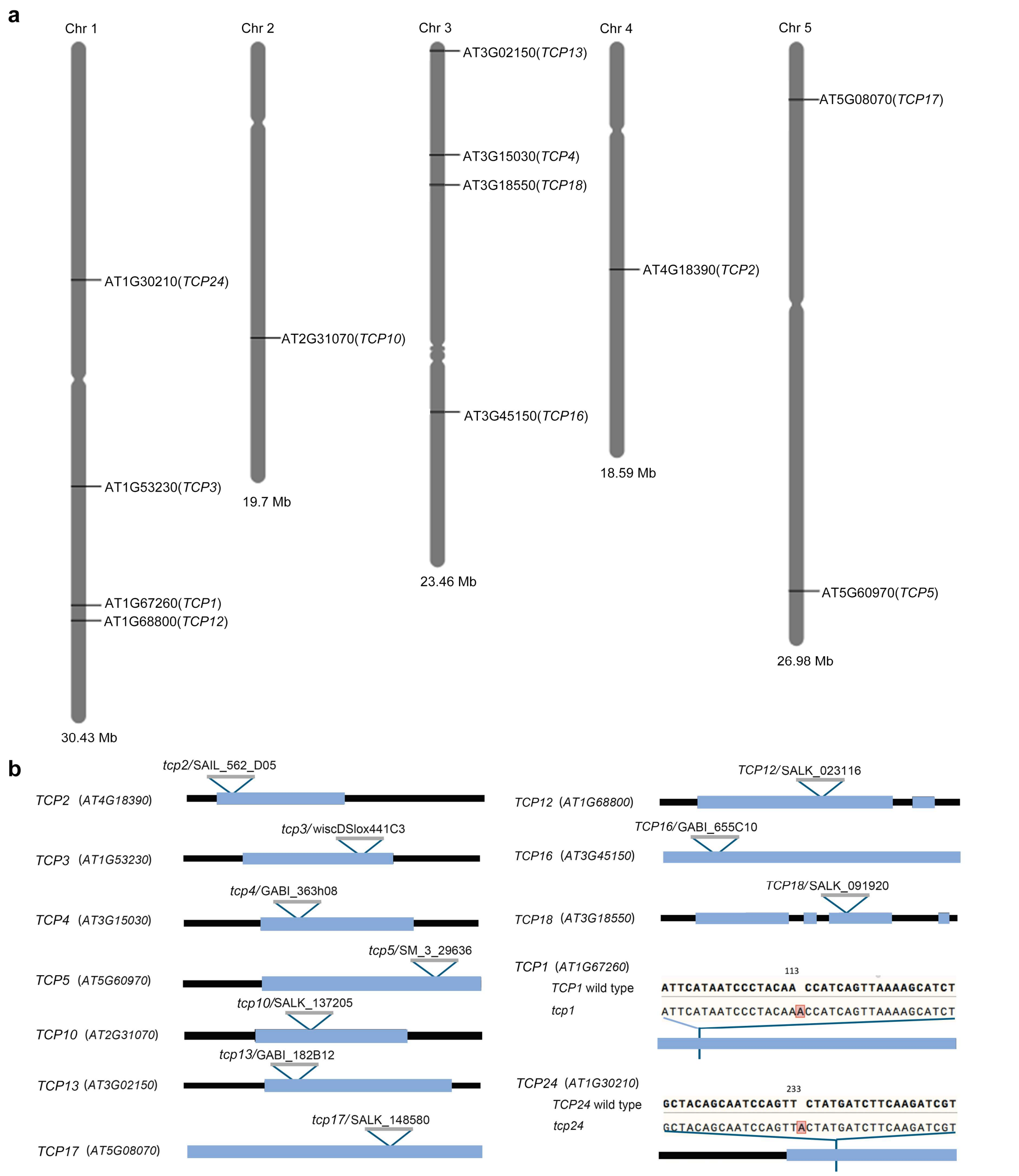

Supplementary Fig. 1 Distribution of *TCP* genes in Arabidopsis chromosomes and a schematic diagram of *tcp* mutants used for the generation of *tcpDUO*. a, Physical loci of class II *TCPs* and *TCP16* in chromosomes. **b**, Schematic diagrams of T-DNA inserts in *tcp* insertion mutants and point mutations in *tcp1* and *tcp24* generated by CRISPR/Cas9 technology.

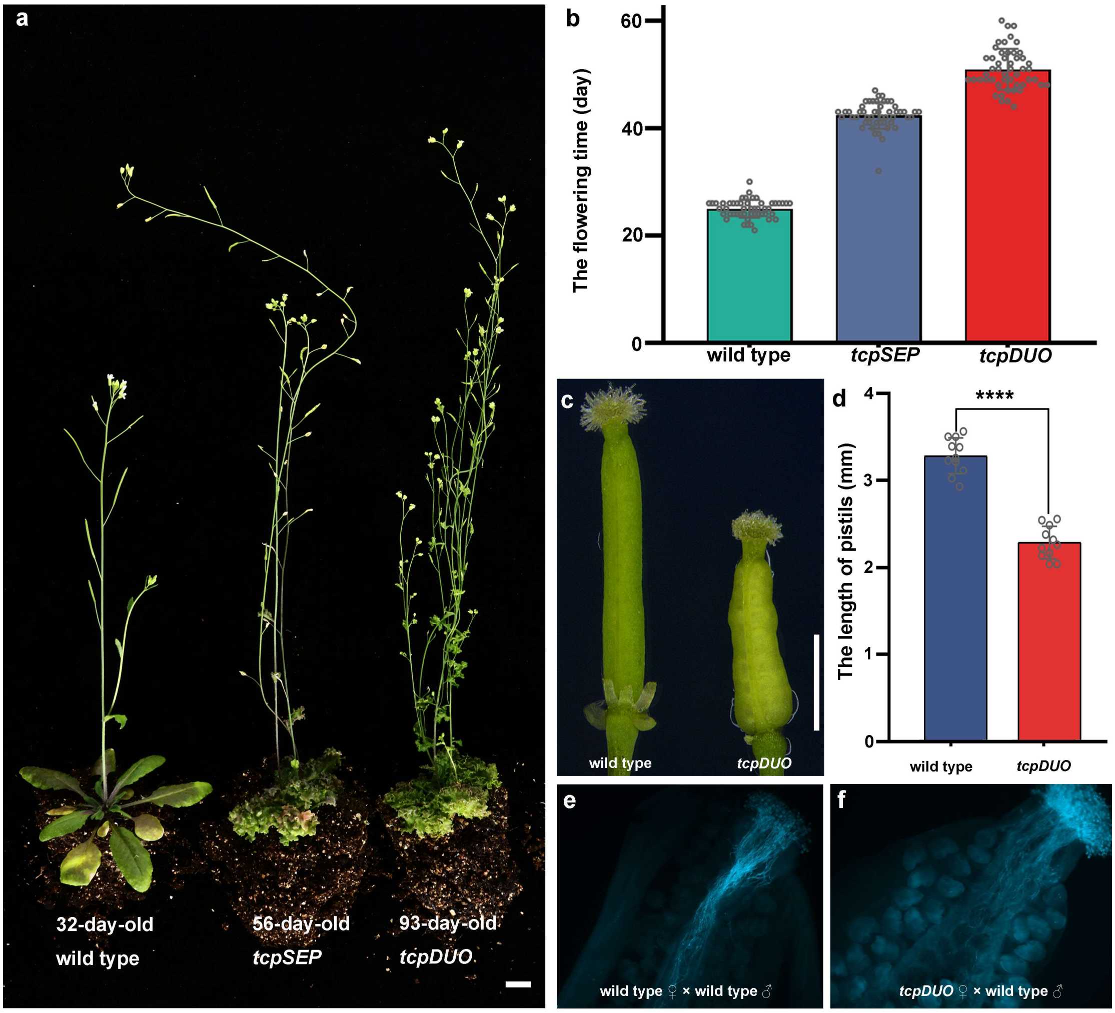

Supplementary Fig. 2 The *tcpDUO* exhibited defects during vegetative and reproductive development. **a**, The mature plants of wild type, *tcpSEP* and *tcpDUO*. **b**, The statistical analysis of the flowering time in wild-type, *tcpSEP* and *tcpDUO*. **c**, The length of pistil of *tcpDUO* was shorter than that in wild type by the observation with a dissecting microscope. **d**, The statistical analysis of the length of pistils from wild type and *tcpDUO*. Student's t-test was used for the analysis of significance. n=11, ****, $P < 0.0001$. Source data are provided as a Source Data file. **e** and **f**, The staining with aniline blue of pollen tubes in wild type (**e**) and *tcpDUO* (**f**). Scale bars, 1 cm in **a,** and 1 mm in **c**.

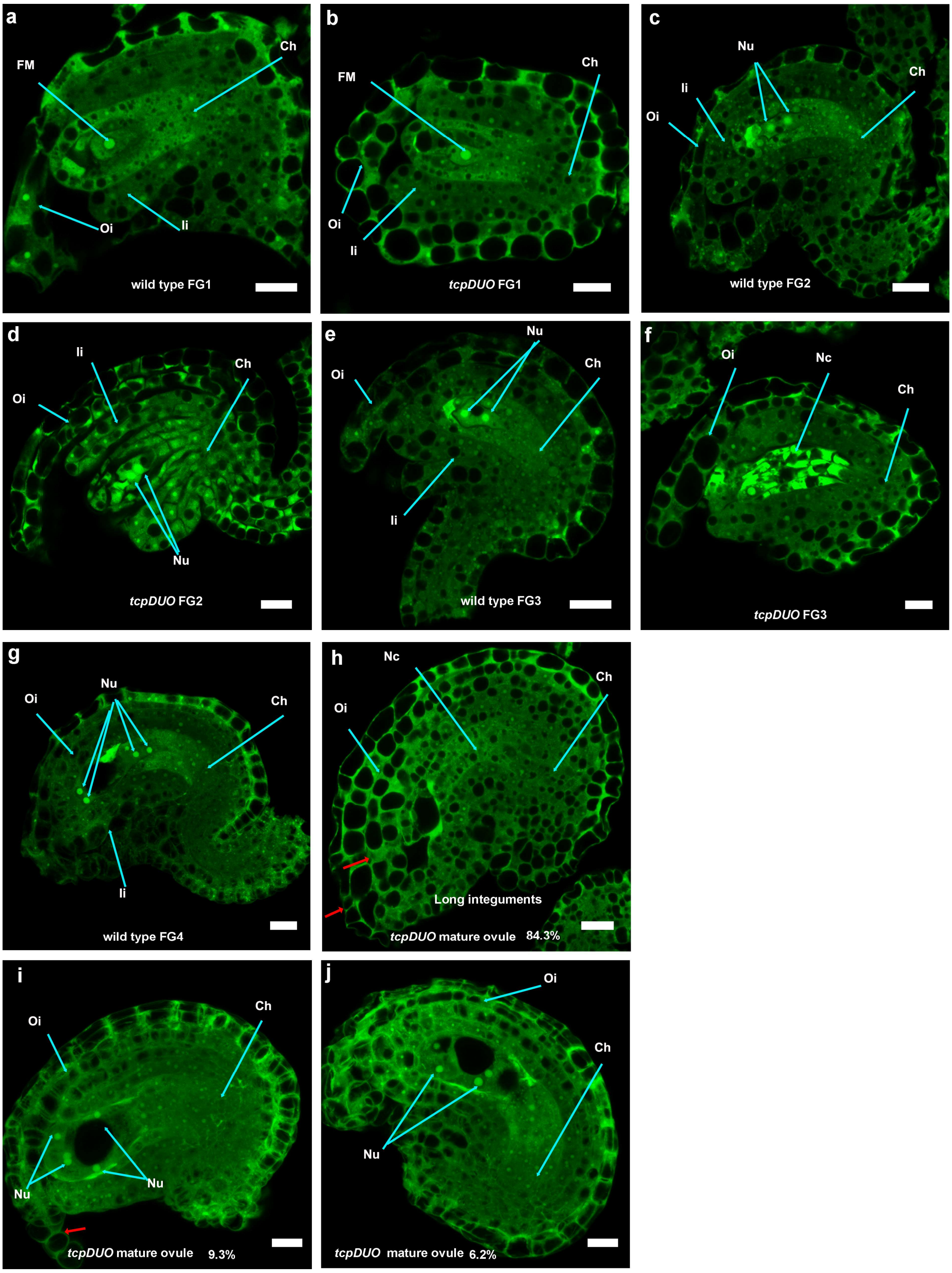

Supplementary Fig. 3 Autofluorescence of ovules during megagametogenesis in wild-type and *tcpDUO*. a-j, Confocal laser scanning microscope images of wild-type (**a**, **c**, **e**, **g**) and *tcpDUO* (**b**, **d**, **f**, **h**, **i**, **j**) at FG1 (**a**, **b**), FG2 (**c**, **d**), FG3 (**e**, **f**), FG4 and subsequent mature stages (**g**, **h**, **i**, **j**). FM, functional megaspore. Ch, chalaza. Nc, nucellus. Ii, inner integument. Oi, outer integuments. Nu, nucleus. Scale bars, 10 µm in **a**-**j**. Red arrows indicate the longer integuments.

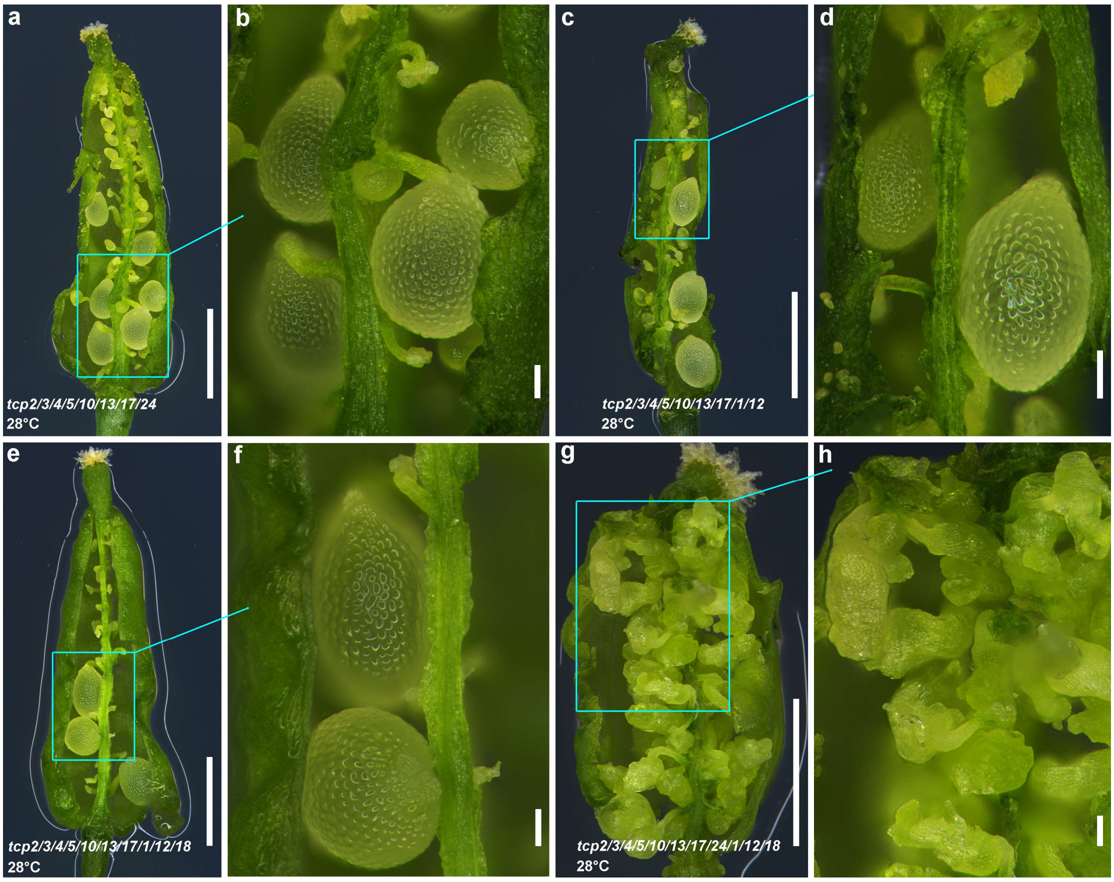

Supplementary Fig. 4 The observation of homeotical conversion of different *tcp* multiple mutants under 28°C treatment using a dissecting microscope. a and **b**, The mature ovules of *tcp2/3/4/5/10/13/17/24* under HT. **c** and **d**, The mature ovules of *tcp2/3/4/5/10/13/17/1/12* under HT. **e** and **f**, The mature ovules of *tcp2/3/4/5/10/13/17/1/12/18* under HT. **g** and **h**, Disruption of Class II TCP transcription factors in *tcp2/3/4/5/10/13/17/24/1/12/18* caused all the ovules to be converted into carpelloid structures under HT. Scale bars, 1 mm in **a**, **c**, **e**, and **g**, and 0.1 mm in **b**, **d**, **f**, and **h**.

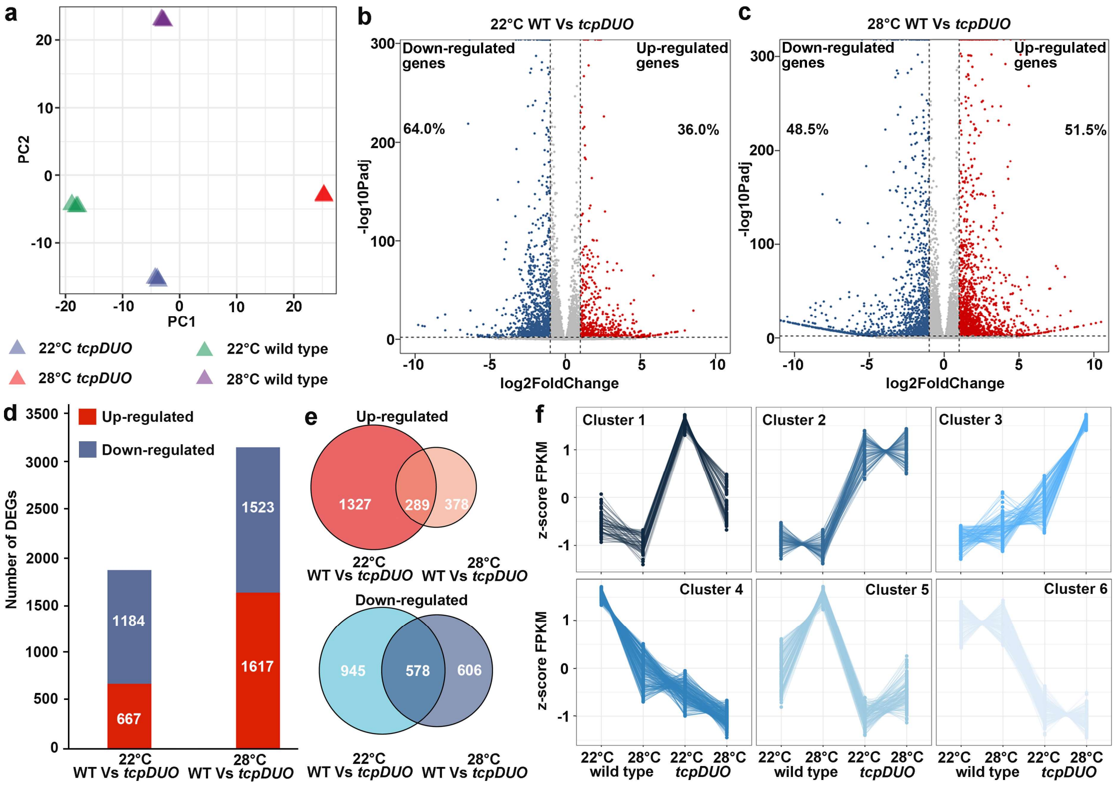

Supplementary Fig. 5 RNA-seq analysis of the pistils from wild-type or *tcpDUO* under 22°C or 28°C. a, Principle component analysis (PCA) of RNA-seq with three replicates. **b,** Volcano plot of the expressed genes in the pistils from wild-type and *tcpDUO* under 22°C. **c,** Volcano plot of the expressed genes in wild-type and *tcpDUO* under 28°C. The threshold of DEGs was Padj < 0.01, fold-change ≥ 2 or ≤ -2. **d**, DEGs (fold change ≥ 2.0 or ≤ -2.0 and false discovery < 0.01) between the pistils from wild-type and *tcpDUO* under 22°C and 28°C. **e**, Overlapping DEGs between WT Vs *tcpDUO* under 28°C and 22°C. **f**, Cluster analysis of the overlapping DEGs between WT Vs *tcpDUO* under 28°C or 22°C.

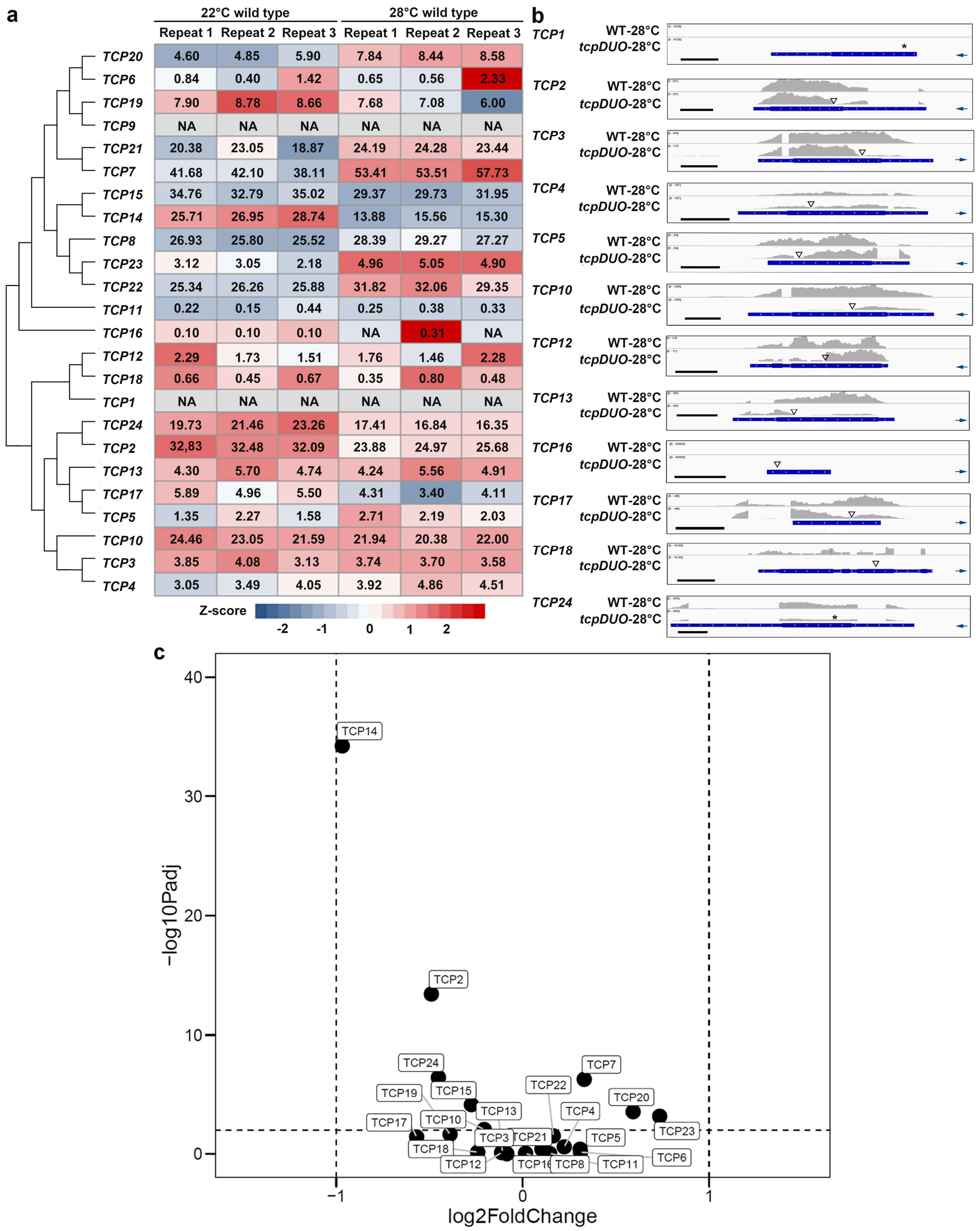

Supplementary Fig. 6 The expression levels of *TCP* family genes. a, The expression levels of *TCP* genes in pistils based on the RNA-seq data of wild-type under 22°C or 28°C. The FPKM (fragments per kilo base of transcript per million mapped fragments) of *TCP* genes in wild-type pistils was standardized. **b**, Mapping of the class II *TCPs* and *TCP16* reads from wild-type and *tcpDUO* under 28°C. The triangles on the peak charts indicate the T-DNA insertion sites. The asterisks indicate the point mutation sites generated by CRISPR/Cas9. Precise mutation sites are listed in Supplementary Figure 1. The blue arrows alongside the gene structure diagrams indicate the transcriptional directions. **c**, The expression fold change of *TCP* genes in wild type treated with 28°C Vs 22°C from RNA-seq data.

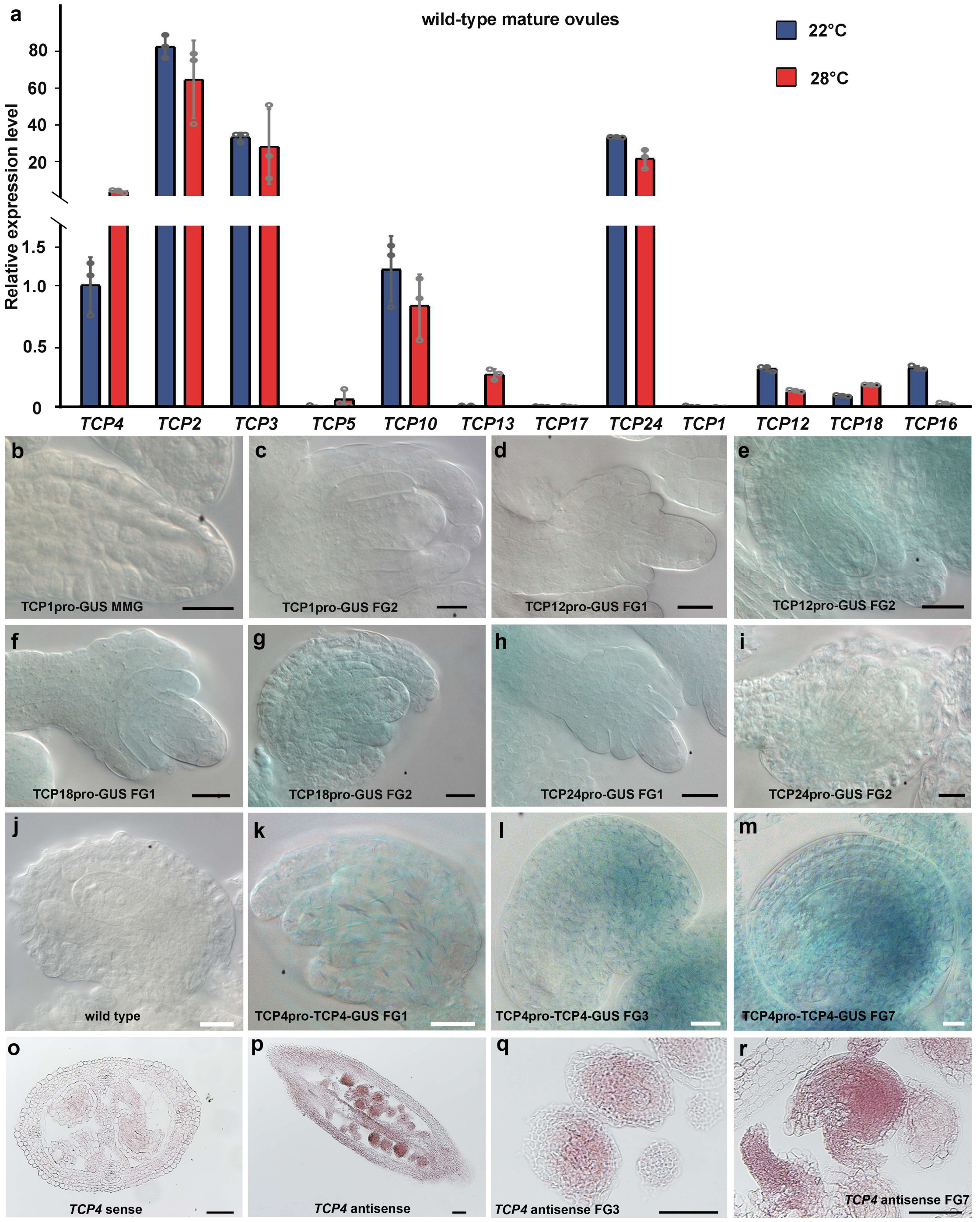

Supplementary Fig. 7 The expression patterns of *TCP* family genes. a, The expression levels of 12 *TCP* genes in mature ovules using RT-qPCR analysis. Source data are provided as a Source Data file. **b** and **c**, The GUS staining of TCP1pro-GUS showed that *TCP1* was hardly detected in ovules. **d** and **e**, The GUS staining of TCP12pro-GUS showed that *TCP12* started to be expressed in ovules at FG2. **f** and **g**, The GUS staining of TCP18pro-GUS showed that *TCP18* was expressed in early ovules. **h** and **i**, The GUS staining of TCP24pro-GUS showed that *TCP24* was expressed in early ovules. **j-m**, GUS staining of ovules from a TCP4pro-TCP4-GUS (**k-m**) and the wild-type control (**j**) showed that TCP4 was expressed during ovule development. **o-r**, In situ hybridization indicated that TCP4 was expressed in ovules. Scale bars, 20 μm in **b-r**.

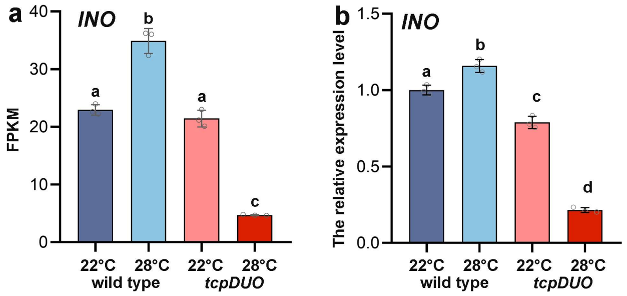

Supplementary Fig. 8 ***INO*** **key for ovule development was down-regulated in** ***tcpDUO*** **under HT. a**, The FPKM from RNA-seq data indicated that *INO* was down-regulated in *tcpDUO* under HT. **b**, The RT-qPCR using mature ovules confirmed that *INO* was down-regulated in *tcpDUO* under HT. Source data are provided as a Source Data file.

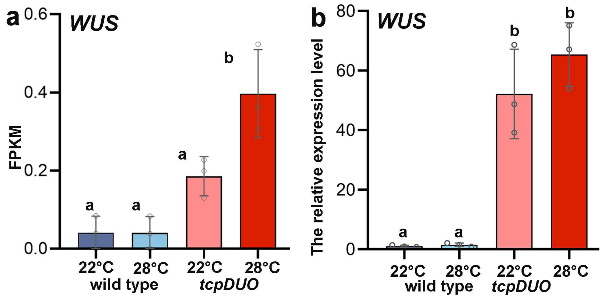

Supplementary Fig. 9 ***WUS*** **was up-regulated in the ovules of** ***tcpDUO*** **under normal temperature or HT. a**, The FPKM from RNA-seq data indicated that *WUS* was induced in pistils of *tcpDUO* under HT. **b**, The RT-qPCR analysis using mature ovules indicated that *WUS* was up-regulated in *tcpDUO* under normal temperature or HT. Source data are provided as a Source Data file.

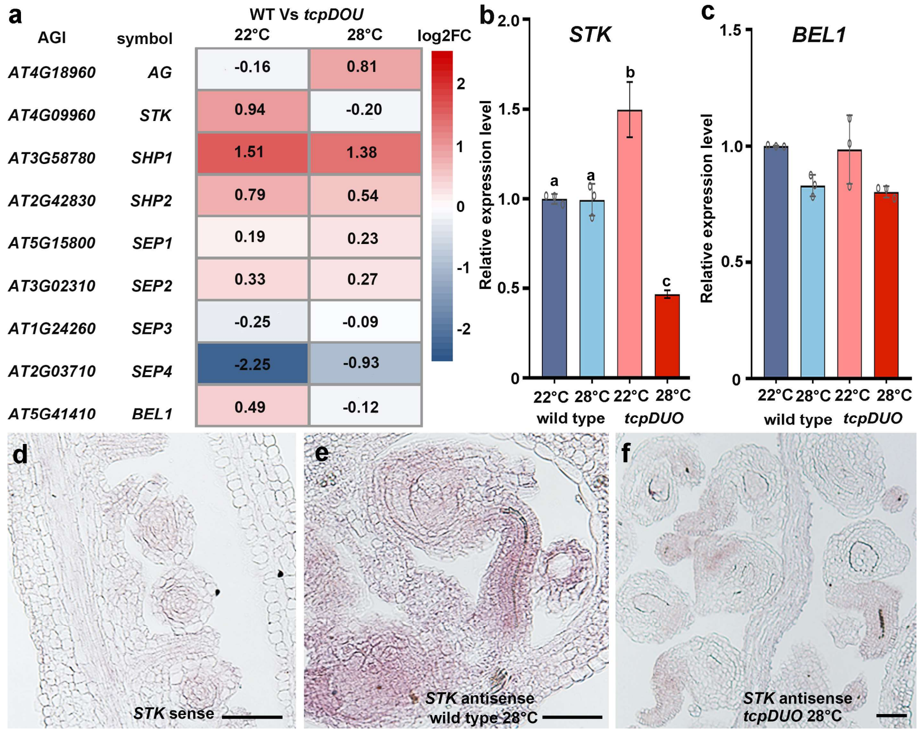

Supplementary Fig. 10 The expression levels of carpel- or ovule-related genes in the pistils of wild-type or *tcpDUO* under 22°C or 28°C. a, Log2 fold-change of the carpel- and ovule-related genes in the comparison of *tcpDUO* with wild-type. The data were from RNA-seq analysis. **b** and **c**, The relative expression level of *STK* (**b**) and *BEL1* (**c**) in mature ovules from wild-type and *tcpDUO* under 22°C or 28°C was determined by RT-qPCR. The expression levels of *STK* or *BEL1* in the wild-type pistils under 22°C were set as 1.0. The data are the mean (±SD) of three biological replicates. One-way ANOVA analysis of variance was performed with LSD/Duncan pairwise comparison testing. Different lowercase letters indicate significant differences ($P < 0.01$). Source data are provided as a Source Data file. **d-f**, In situ hybridization suggested that the transcripts of *STK* were decreased in *tcpDUO*. Scale bars, 20 µm in d-f.

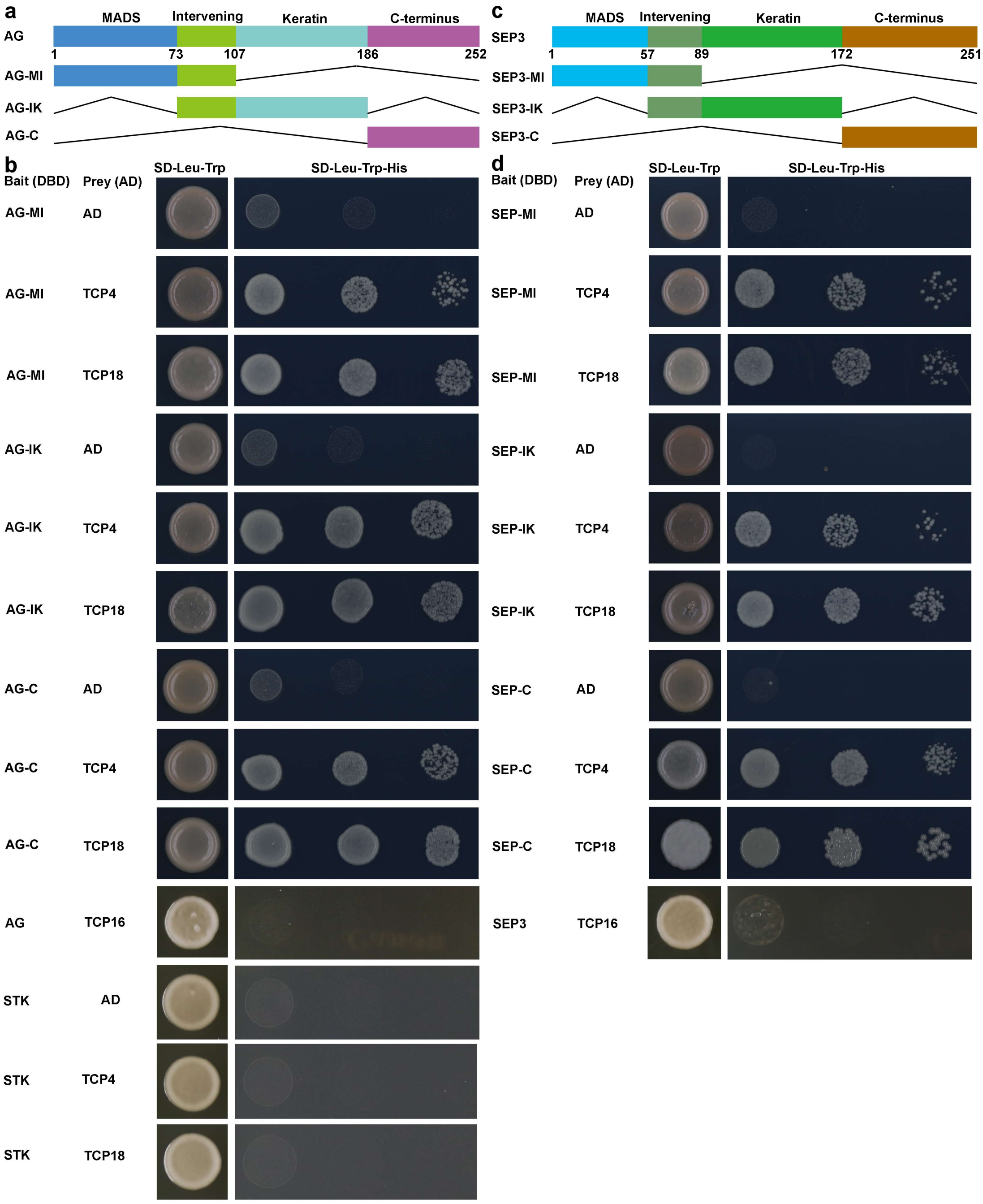

Supplementary Fig. 11 Deletion analysis of the interaction between AG or SEP3 and TCP4 or TCP18. **a**, Schematic diagrams of AG and its truncations. **b**, The yeast-two-hybrid assays showed that all AG truncations interacted with TCP4 or TCP18, while no interactions were detected in the negative controls. The AG, AG truncations and STK were used as bait, and TCP4, TCP18 or TCP16 was used as prey. **c**, Schematic diagrams of SEP3 and its truncations. **d**, The yeast-two-hybrid assays showed that all SEP3 truncations interacted with TCP4 or TCP18, while no interaction was found in SEP3 and TCP16. The SEP3 truncations were used as bait, and TCP4, TCP18 or TCP16 was used as prey. The transformed yeasts were spotted on the selection SD-Leu-Trp-His medium with 10-, 100-, and 1,000-fold dilutions. 2.5 mM 3-amino-1,2,4-trizole was supplemented to inhibit the self-activation activity of the bait. AD, activation domain; DBD, DNA-binding domain.

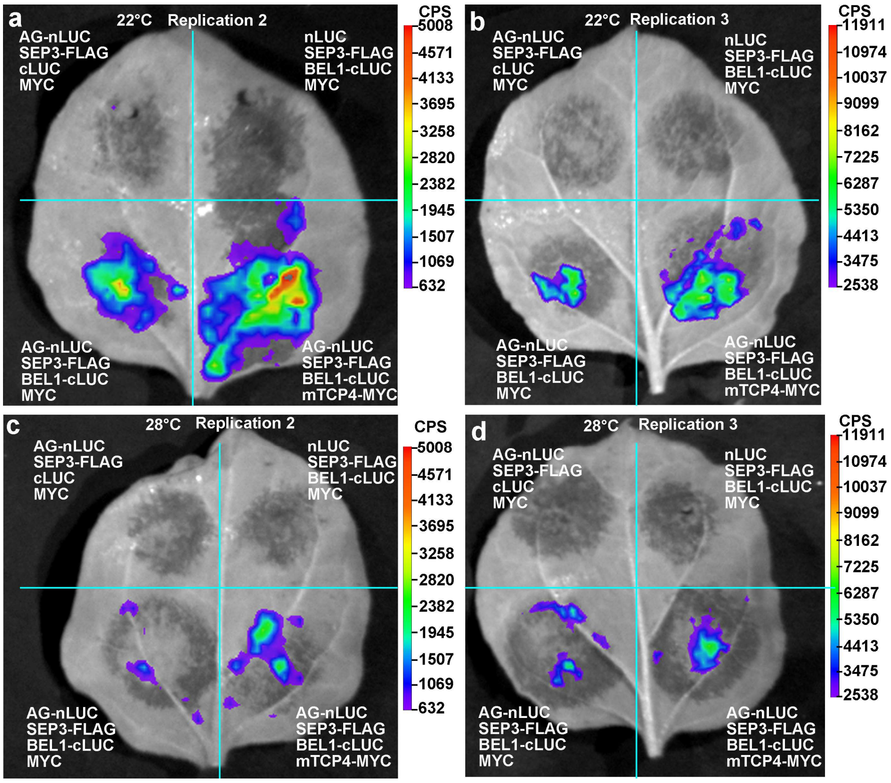

Supplementary Fig. 12 The two replications of the experiments in Fig. 6d and 6e. **a** and **b**, The two replications showed that the association of BEL1 with AG-SEP3 was enhanced by the co-expression of miR319-resistant *mTCP4* under 22°C using a transient expression system in tobacco leaves. **c** and **d**, The two replications showed that the association of BEL1 with AG-SEP3 was obviously decreased under HT.

Supplementary Table 8. Primers used in this study.

**Genotype analysis**	
**Primers**	**Sequences 5′→ 3′**
TCP1-Genotyping-F	TGATTCTTGGTTCTTGATTC
TCP1-Genotyping-R	GCTTGTACGACCTCTTTGAT
TCP2-G-F	AACCCGTTTTATCAATTGTTGTG
TCP2-T-F	TAGCATCTGAATTTCATAACCAATCTCGATAC
TCP2-G-R	AACCGGAATTTAACAAATCCG
TCP3-G-F	ACCAAGCACGAATCATAGGTG
TCP3-T-F	AACGTCCGCAATGTGTTATTAAGTTGTC
TCP3-G-R	TTTAGGGTTTGGGATTTGGAG
TCP4-G-F	GGAACGATTGCAGCGAGAGA
TCP4-T-F	GGGCTACACTGAATTGGTAGCTC
TCP4-G-R	TCTGGGTTTTTCTTGATTGGTCA
TCP5-G-F	TGAATCTGTTTTTCCTCCATCC
TCP5-T-F	TACGAATAAGAGCGTCCATTTTAGAGTGA
TCP5-G-R	CTCGAAGCAGCAAAAGATGAC
TCP10-G-F	AAGCAATGTCATCATCGACGG
TCP10-T-F	ATTTTGCCGATTTCGGAAC
TCP10-G-R	ACGGCAAGCATTTTGAAGAGG
TCP12-G-F	GCTATTTGGCTATCCTGAGGC
TCP12-T-F	ATTTTGCCGATTTCGGAAC
TCP12-G-R	AAGTGTGTTGATGGAAGCACC
TCP13-G-F	ATGAATATCGTCTCTTGGAAAGA
TCP13-T-F	GGGCTACACTGAATTGGTAGCTC
TCP13-G-R	TGATGATGTTCCAGGTTGTGA
TCP16-G-F	ATTGATAGTCCGGGGAATTTG
TCP16-T-F	GGGCTACACTGAATTGGTAGCTC
TCP16-G-R	AAGCTCAAACTGTGGTTGTGG
TCP17-G-F	TCTTTGGATCCTCAGATCTTCC
TCP17-T-F	ATTTTGCCGATTTCGGAAC
TCP17-G-R	ATGTACCTTTGCTCGCATCAG
TCP18-G-F	TGTAGAACAACCCACTGAGCC
TCP18-T-F	ATTTTGCCGATTTCGGAAC

TCP18-G-R	ATCGATGGTGGTGCATTAGTG
TCP24-Genotyping-F	TAGCGTTACAATGGAGGTTG
TCP24-Genotyping-R	GCATTCTGCAAATCTTTATCTC
BEL1-Genotyping -F	GATCACCTGTCTTCTGATCTCTGAAG
BEL1-Genotyping -R	AGTGAAGTGGTGGAACATGTTTCT
STK-Genotyping -F	GAAGGATGGGAAGAGGAAAGATAG
STK-Genotyping -R	CCTGTTGGAGTTTTGAATCGTTTG

Cloning primers

Primers	Sequences 5′→ 3′
TCP4-F	ATGTCTGACGACCAATTCCATCAC
TCP4-sc-R	TCAATGGCGAGAAATAGAGGAAGCAG
TCP4-nsc-R	ATGGCGAGAAATAGAGGAAGCAGA
TCP4-pro-F	GAAACATGATTCCAACTTGTGCATG
TCP16-CDS-F	ATGGATTCGAAAAATGGAATTAACAACA
TCP16-CDSsc-R	TCAAACTGTGGTTGTGGCTGT
TCP18-CDS-F	ATGAACAACAACATTTTCAGTACTACTAC
TCP18-CDSsc-R	TCAATACATGTTTTGATAGTTGTGCATGA
AG-CDS-F	ACGGCGTACCAATCGGAG
AG-CDSsc-R	TTACACTAACTGGAGAGCGGTTTGG
AG-CDSnsc-R	CACTAACTGGAGAGCGGTTTGG
STK-CDS-F	ATGCTCTTTCCCCATGAAAGAAAGAAAG
STK-CDSsc-R	TTATCCGAGATGAAGAATTTTCTTGTCGG
BEL1-CDS-F	ATGGCAAGAGATCAGTTCTATGGTCA
BEL1CDSsc-R	TCAAACAATATCATGAAGTAATTGAGC
BEL1-CDSnsc-R	AACAATATCATGAAGTAATTGAGCTCCCA
SEP3-CDS-F	ATGGGAAGAGGGAGAGTAGAATTGAAG
SEP3-CDSsc-R	TCAAATAGAGTTGGTGTCATAAGGTAACC
CRCpro-F	TCCCTTTGTCTATTCGCAGTTGTAAAGT
CRCpro-R	GGTCTTTAGCGAATGGATTGAAAACCG
SPTpro-5K-F	AGGTGTTTTCACATCCCAACATG
SPTpro-R	TACACCAACAACAAAAAAAAAGCAG
AG-I-FC-F	TCTAACAACAGTGTAAAAGGG
AG-I-FC-R	TTATTGATAATACTGTGCATTAATTTC
AG-K-FC-F	CAAGAATCAGCCAAATTGCG

AG-K-FC-R	TTACTTTGCACGAAGAATCTGGT
AG-M-FC-R	TTAGTACTCATAGAGACGACCACGG
AG-C-FC-F	ATAGCTGAAAATGAGAGGAAC
SEP3-I-FC-F	TGCAGTAGTTCGAGCATGC
SEP3-I-FC-R	TCAAACTGCTAAGGCCTCTCTTG
SEP3-K-FC-F	GAACTTAGTAGCCAGCAGGAG
SEP3-K-FC-R	TCACCTTAGTCTTAGAGTTTTATTTGT
SEP3-M-FC-R	TCAAAACTCGTACAGCTTTCCTCTATT
SEP3-C-FC-F	TTAGCTGATGGGTATCAGATGCC
Gib-BEL1-Flag-F	CCGCGGCCGCCCCCTTCACCATGGCA AGAGATCAGTTCTATGG
Gib-BEL-Flag-R	CAATATCATGATCCTTGTAGTCTCCGT CGTGGTCCTTATAGTCAACAATATCATGA AGTAATTG
Gib-P2A-GFP-F	CTACAAGGATCATGATATTGATTACA AAGACGATGACGATAAGAAGCTTGGAAG CGGAGCTAC
Gib-P2A-GFP-R	GGCGCGCCCACCCTTTTACTTGTACA GCTCGTCCATG

CRISPR/Cas9 sgRNA clone primers

Primers	Sequences 5′→ 3′
BEL1-sg1-F	GATTGTCTATCATCATACCCATGT
BEL1-sg1-R	AAACACATGGGTATGATGATAGAC
BEL1-sg2-F	GATTTCACTCAGTAGCTCTTGAGC
BEL1-sg2-R	AAACGCTCAAGAGCTACTGAGT
STK-sg1-F	GATTATTGGCGTATTCATAGAGA
STK-sg1-R	AAACTCTCTATGAATACGCCAAT
STK-sg2-F	GATTTCTTGGACAGTGCTAGTGT
STK-sg2-R	AAACACACTAGCACTGTCCAAGA

Gene expression analysis

Primers	Sequences 5′→ 3′
ACT7-RT-F	TCGGAGCTGAGAGATTCCGT
ACT7-RT-R	TGGAACCACCACTGAGAACG

CRC-RT-F	CTTCCCCTCAAGCCGAACAT
CRC-RT-R	AAGGGTGAGGCTAACATGGC
SPT-RT-F	CCCCCTGAAGCAACCAATGA
SPT-RT-R	CTTCCCGACTCATCTCCACG
HEC1-RT-F	ACGAACCAGGTTTCCGCTAC
HEC1-RT-R	CATCACGGCGATACGGAAGA
HEC2-RT-F	AGCCGTGATGCAGCCTATAC
HEC2-RT-R	TCTTAGTCCCACCGGGAACA
INO-RT-F	TGCCATGTCCAGTGTGGTTT
INO-RT-R	CAACCTCCTCTTTCCCGGTc
WUS-RT-F	TCACTGCAAGGCTGAGACAG
WUS-RT-R	TAGCCGCCATCATAACCGAG
STK-RT-F	TCACCAGATTAAGAATTAGGATGGG
STK-RT-R	TCATAGAGACGGCCACGAGT
BEL-RT-F	TTGCTCGATCAAGCTTTGCG
BEL-RT-R	TAAACCAGTTTGTCGGGCCA
TCP1-RT-F	ACCAACGAAGATGGGTCAAAGT
TCP1-RT-R	TGAATCCAAACTTTACCTTTGGGG
TCP2-RT-F	CGTCGTGTCCGGTTATCAGT
TCP2-RT-R	TGATTCTCGTCATCGGTCGG
TCP3-RT-F	TCCGACGGCGATTCAATTCT
TCP3-RT-R	TGGGTTTAGCGTTAGCAGCA
TCP4-RT-null-F	AAAGTCTGCACGGCTAAAGG
TCP4-RT-null-R	GGCTAGGCGAATTGCATCGG
TCP5-RT-null-F	CAGCTTATTACAACCTTGGACATC
TCP5-RT-null-R	GGGAATAGAGAGCTCATTGCC
TCP10-RT-null-F	CTACTCGTCAAGAACCGGTG
TCP10-RT-null-R	CCTGATGAGGGTAGTTTTGGAAAT
TCP12-RT-null-F	GGATGAGACACTGAAGTTGAGAG
TCP12-RT-null-R	CTCTCTAGTTTCTTGATGAGGATCTG
TCP13-RT-F	TGTCCAATGACGACATCACCA
TCP13-RT-R	GTCGAAATGTTTTGGGAAGACGA
TCP16-RT-F	AGTTGGCTCCTCCAGAATGC
TCP16-RT-R	CCGGAAACACCATCTGACGA

TCP17-RT-null-F	TGATCACATCGATCATCACAACC
TCP17-RT-null-R	GAAAATGCGACAGTGACGTT
TCP18-RT-F	GACTCTCGCTAGATGTCGCC
TCP18-RT-R	CTGAAGCAGCCATGGTGAGA
TCP24-RT-F	TGAGAGTTCGCTGTTGTCGT
TCP24-RT-R	TCCGGTTACTCGGTTGTTGG

EMSA primers

Primers	Sequences 5′→ 3′
CRC-EMSA-F	CAGGAAAGGACCATTCTTAA
CRC-EMSA-R	TTAAGAATGGTCCTTTCCTG
mCRC-EMSA-F	CAGGAAAAAAAAATTCTTAA
mCRC-EMSA-R	TTAAGAATTTTTTTTTCCTG
1-SPT-EMSA-F	GAAAATCTCGTGGTCCATCTAAAAAT
1-SPT-EMSA-R	ATTTTTAGATGGACCACGAGATTTTC
1m-SPT-EMSA-F	GAAAATCTCAAAAAAAATCTAAAAAT
1m-SPT-EMSA-R	ATTTTTAGATTTTTTTTGAGATTTTC
2-SPT-EMSA-F	CGTCTTTATGGTCCATTCTATATG
2-SPT-EMSA-R	CATATAGAATGGACCATAAAGACG
2m-SPT-EMSA-F	CGTCTTTAAAAAAAATTCTATATG
2m-SPT-EMSA-R	CATATAGAATTTTTTTTAAAGACG

***in situ* primers**

Primers	Sequences 5′→ 3′
TCP4-insitu-T7-F	TAATACGACTCACTATAGGGTATACAGGAAACGGAGGAGGGT
TCP4-insitu-R	TTGTGAGTGAGACCGTCGTG
TCP4-insitu-F	ATACAGGAAACGGAGGAGGGT
TCP4-insitu-T7-R	AATTAATACGACTCACTATAGGGTTGTGAGTGAGACCGTCGTG
STK-insitu-T7-F	TAATACGACTCACTATAGGGGATCCAAACGATTCAAAACT
STK-insitu-R	GAGATGAAGAATTTTCTTGTC
STK-insitu-F	AGATCCAAACGATTCAAAACT
STK-insitu-T7-R	TAATACGACTCACTATAGGGGAGATGAAGAATTTTCTTGTC
