# Supplementary material for: Arabidopsis TCP4 transcription factor inhibits high temperature-induced homeotic conversion of ovules
Source: Nat Commun. 2023 Sep 13;14:5673. doi: 10.1038/s41467-023-41416-1 (PMC10499876; doi:10.1038/s41467-023-41416-1)
Supplement: Supplementary file 3 — Description of Additional Supplementary Files [file 41467_2023_41416_MOESM3_ESM.pdf]

Description of Additional Supplementary Files

Supplementary Data 1. Genes up-regulated in tcpDUO mutant under 22℃

Supplementary Data 2. Genes down-regulated in tcpDUO mutant under 22℃

Supplementary Data 3. Genes up-regulated in tcpDUO mutant under 28℃

Supplementary Data 4. Genes down-regulated in tcpDUO mutant under 28℃

Supplementary Data 5. Genes in the Co-expression Clustering Analysis

Supplementary Data 6. GO Analysis of the Genes in Cluster 3

Supplementary Data 7. GO Analysis of the Genes in Cluster 4
