# Supplementary material for: Arabidopsis TCP4 transcription factor inhibits high temperature-induced homeotic conversion of ovules
Source: Nat Commun. 2023 Sep 13;14:5673. doi: 10.1038/s41467-023-41416-1 (PMC10499876; doi:10.1038/s41467-023-41416-1)
Supplement: Supplementary file 11 — Reporting Summary [file 41467_2023_41416_MOESM11_ESM.pdf]

nature portfolio

Corresponding author(s): Genji Qin

Last updated by author(s): Aug 25, 2023

Reporting Summary

Nature Portfolio wishes to improve the reproducibility of the work that we publish. This form provides structure for consistency and transparency in reporting. For further information on Nature Portfolio policies, see our Editorial Policies and the Editorial Policy Checklist.

Statistics

For all statistical analyses, confirm that the following items are present in the figure legend, table legend, main text, or Methods section.

n/a	Confirmed	
☐	☒	The exact sample size (*n*) for each experimental group/condition, given as a discrete number and unit of measurement
☐	☒	A statement on whether measurements were taken from distinct samples or whether the same sample was measured repeatedly
☐	☒	The statistical test(s) used AND whether they are one- or two-sided<br>*Only common tests should be described solely by name; describe more complex techniques in the Methods section.*
☒	☐	A description of all covariates tested
☐	☒	A description of any assumptions or corrections, such as tests of normality and adjustment for multiple comparisons
☐	☒	A full description of the statistical parameters including central tendency (e.g. means) or other basic estimates (e.g. regression coefficient) AND variation (e.g. standard deviation) or associated estimates of uncertainty (e.g. confidence intervals)
☐	☒	For null hypothesis testing, the test statistic (e.g. *F*, *t*, *r*) with confidence intervals, effect sizes, degrees of freedom and *P* value noted<br>*Give P values as exact values whenever suitable.*
☒	☐	For Bayesian analysis, information on the choice of priors and Markov chain Monte Carlo settings
☐	☒	For hierarchical and complex designs, identification of the appropriate level for tests and full reporting of outcomes
☒	☐	Estimates of effect sizes (e.g. Cohen's *d*, Pearson's *r*), indicating how they were calculated

Our web collection on statistics for biologists contains articles on many of the points above.

Software and code

Policy information about availability of computer code

Data collection	We used ZEN and LAS software to collect images on the microscope. We used indIGO software to collect in planta fluorescence images.
Data analysis	We used Tanon, ZEN, LAS and indiGO software for data analysis, Microsoft Excel and prism 9 for statistical analysis.

For manuscripts utilizing custom algorithms or software that are central to the research but not yet described in published literature, software must be made available to editors and reviewers. We strongly encourage code deposition in a community repository (e.g. GitHub). See the Nature Portfolio guidelines for submitting code & software for further information.

Data

Policy information about availability of data

All manuscripts must include a data availability statement. This statement should provide the following information, where applicable:

- Accession codes, unique identifiers, or web links for publicly available datasets
- A description of any restrictions on data availability
- For clinical datasets or third party data, please ensure that the statement adheres to our policy

The RNA sequencing raw data have been deposited and available in the Sequence Read Archive (SRA) under BioProject ID PRJNA891376 (https://www.ncbi.nlm.nih.gov/bioproject/PRJNA891376). TCP4 ChIP-seq data are available under BioProject ID PRJNA474688 (https://www.ncbi.nlm.nih.gov/bioproject/474688). The Arabidopsis reference genome used in this article is TAIR10 (https://www.arabidopsis.org/). Source data are provided with this paper.

Human research participants

Policy information about studies involving human research participants and Sex and Gender in Research.

Reporting on sex and gender	N/A
Population characteristics	N/A
Recruitment	N/A
Ethics oversight	N/A

Note that full information on the approval of the study protocol must also be provided in the manuscript.

Field-specific reporting

Please select the one below that is the best fit for your research. If you are not sure, read the appropriate sections before making your selection.

☒ Life sciences ☐ Behavioural & social sciences ☐ Ecological, evolutionary & environmental sciences

For a reference copy of the document with all sections, see nature.com/documents/nr-reporting-summary-flat.pdf

Life sciences study design

All studies must disclose on these points even when the disclosure is negative.

Sample size	Figure 4: Three biological replicates were carried out. 20~30 pistils were used for each sample in RNA-seq.<br>Figure 5c, f: 3 mL Arabidopsis protoplast were used for Co-IP experiment.<br>Figure 5g-i: Three biological replicates were carried out.<br>Figure 6c: 0.5 g tobacco leaves were used for Co-IP experiment.
Data exclusions	No data were excluded.
Replication	Each experiment was reproduced at least three times, none were found to be not reproducible.
Randomization	Plants were selected randomly for image and protein works.
Blinding	No blinding used.

Reporting for specific materials, systems and methods

We require information from authors about some types of materials, experimental systems and methods used in many studies. Here, indicate whether each material, system or method listed is relevant to your study. If you are not sure if a list item applies to your research, read the appropriate section before selecting a response.

Materials & experimental systems

n/a	Involved in the study	
☐	☒	Antibodies
☒	☐	Eukaryotic cell lines
☒	☐	Palaeontology and archaeology
☒	☐	Animals and other organisms
☒	☐	Clinical data
☒	☐	Dual use research of concern

Methods

n/a	Involved in the study	
☒	☐	ChIP-seq
☒	☐	Flow cytometry
☒	☐	MRI-based neuroimaging

Antibodies

Antibodies used	The anti-FLAG (Sigma, A8592) antibody<br>The anti-GFP (ABclonal, AE011) antibody<br>The anti-digoxigenin (Roche, 11093274910) antibody
Validation	anti-FLAG (Sigma): https://www.sigmaaldrich.cn/CN/zh/product/sigma/a8592

anti-GFP (ABclonal, AE011) antibody: https://abclonal.com.cn/catalog/AE011
anti-digoxigenin (Roche, 11093274910) antibody: https://www.sigmaaldrich.cn/CN/zh/product/roche/11093274910
